# Strigolactone and karrikin signal perception: receptors, enzymes, or both?

**DOI:** 10.3389/fpls.2012.00296

**Published:** 2012-12-28

**Authors:** Bart J. Janssen, Kimberley C. Snowden

**Affiliations:** Plant Development Team, Breeding and Genomics, Plant & Food Research Institute of New ZealandAuckland, New Zealand

**Keywords:** strigolactone, karrikin, receptor, hormone, branching

## Abstract

The signaling molecules strigolactone (SL) and karrikin are involved in seed germination, development of axillary meristems, senescence of leaves, and interactions with arbuscular mycorrhizal fungi. The signal transduction pathways for both SLs and karrikins require the same F-box protein (MAX2) and closely related α/β hydrolase fold proteins (DAD2 and KAI2). The crystal structure of DAD2 has been solved revealing an α/β hydrolase fold protein with an internal cavity capable of accommodating SLs. DAD2 responds to the SL analog GR24 by changing conformation and binding to MAX2 in a GR24 concentration-dependent manner. DAD2 can also catalyze hydrolysis of GR24. Structure activity relationships of analogs indicate that the butenolide ring common to both SLs and karrikins is essential for biological activity, but the remainder of the molecules can be significantly modified without loss of activity. The combination of data from the study of DAD2, KAI2, and chemical analogs of SLs and karrikins suggests a model for binding that requires nucleophilic attack by the active site serine of the hydrolase at the carbonyl atom of the butenolide ring. A conformational change occurs in the hydrolase that results in interaction with the F-box protein MAX2. Downstream signal transduction is then likely to occur via SCF (Skp-Cullin-F-box) complex-mediated ubiquitination of target proteins and their subsequent degradation. The role of the catalytic activity of the hydrolase is unclear but it may be integral in binding as well as possibly allowing the signal to be cleared from the receptor. The α/β hydrolase fold family consists mostly of active enzymes, with a few notable exceptions. We suggest that DAD2 and KAI2 represent an intermediate stage where some catalytic activity is retained at the same time as a receptor role has evolved.

## SLs AND KARs ARE RELATED PLANT SIGNALING MOLECULES

**FIGURE 1:**
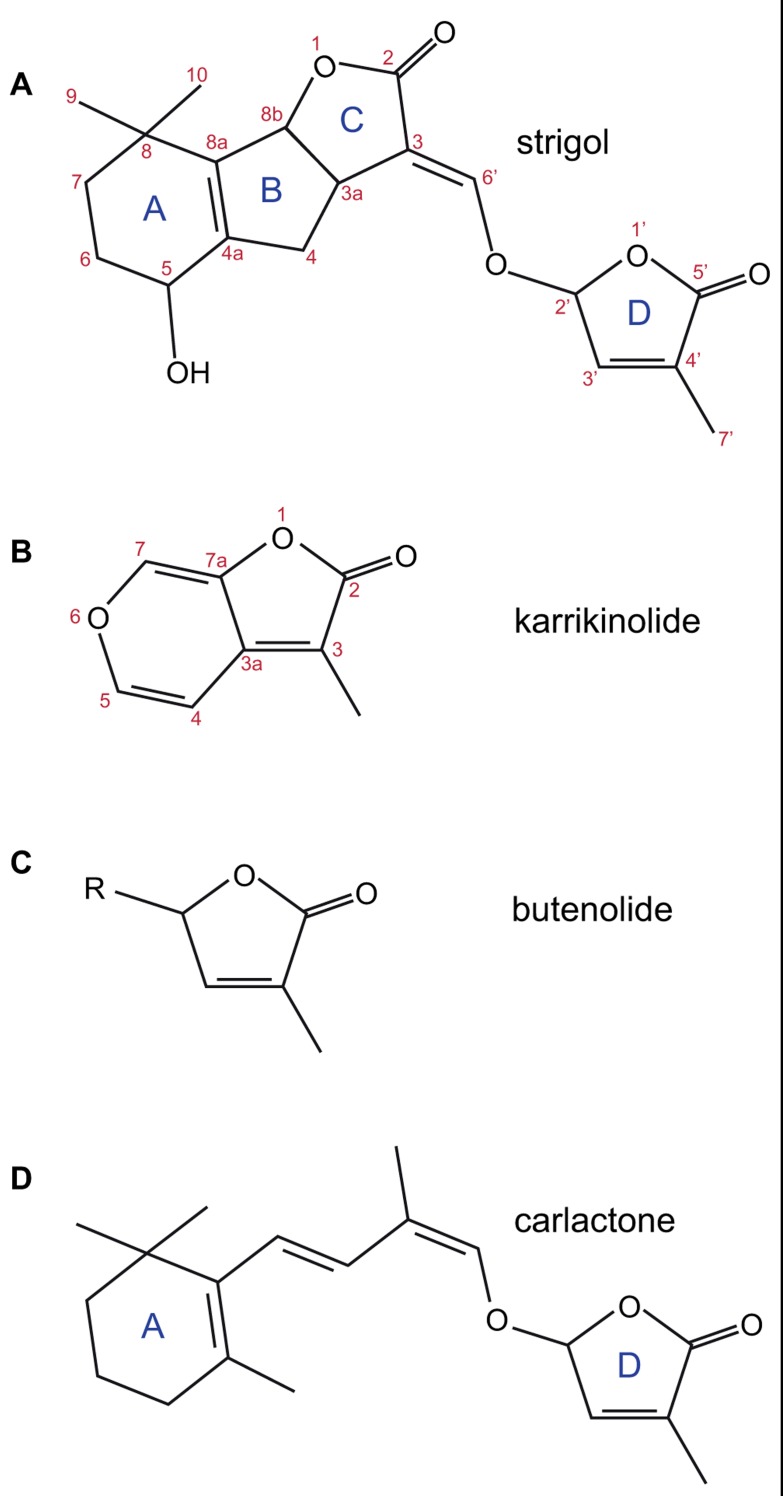
The structures of strigol **(A)**; karrikinolide, KAR_**1**_ **(B)**; the butenolide ring **(C)**; and carlactone **(D)**. The standard lettering of the ring structure in SL is shown as is the standard atom numbering for strigol and karrikin.

Strigolactones (SLs) and karrikins are plant signaling molecules with several common features. Both SLs and karrikins have structures containing a butenolide ring integral to activity and both are involved in seed germination (**Figures 1A**–**C**). While karrikins are not technically hormones, because they are not known to be produced in living cells, for simplicity we will describe both SLs and karrikins as hormones in this review since apart from synthesis, karrikins behave as if they are hormones and in particular the signal reception and transduction pathway operates as a hormone reception pathway.

Strigolactones are a class of compounds produced in the roots of plants (Cook et al., 1966; Siame et al., 1993; Gomez-Roldan et al., 2008; Umehara et al., 2008; Yoneyama et al., 2009; Zwanenburg et al., 2009) particularly under conditions of nutrient stress (Yoneyama et al., 2007a,b; Lopez-Raez et al., 2008; Umehara et al., 2010). Production of SLs may also occur in organs such as stems where the biosynthetic genes are expressed, the role of SL production in these organs is uncertain but may be important. SLs are exuded from the roots, where they act as exogenous signals to stimulate interactions with arbuscular mycorrhizal (AM) fungi, directly stimulating hyphal branching and growth in the symbiotic fungi (Akiyama et al., 2005; Besserer et al., 2006). This symbiotic relationship appears to increase the effective root surface area by using the fungal hyphae to absorb nutrients that are transferred to the plant in exchange for sugars from the plant (Harrison, 2005; Reinhardt, 2007; Parniske, 2008). In the soil SLs are also detected by the seeds of parasitic plants in the *Striga* and *Orobanche* genera, which are significant crop pests worldwide, stimulating germination of these weeds (Cook et al., 1966, 1972; Bouwmeester et al., 2003; Yoneyama et al., 2010).

Strigolactones are also transported within the plant as endogenous signals (Kohlen et al., 2011; Kretzschmar et al., 2012), crossing graft unions to regulate scion development (Napoli, 1996; Foo et al., 2001; Morris et al., 2001; Turnbull et al., 2002; Sorefan et al., 2003; Booker et al., 2004; Snowden et al., 2005; Simons et al., 2007; Drummond et al., 2009a,b). Mutations of the SL biosynthetic or signal transduction pathway result in an increased branching phenotype (*max1*, *2*, *3*, *4*, *Atd14* in Arabidopsis; *dad1*, *2*, *3* in petunia; *rms1*, *2*, *4*, *5* in pea; *d3*, *d10*, *htd1*, *d14* in rice) as well as reduction in plant height and delayed leaf senescence (Arumingtyas et al., 1992; Napoli and Ruehle, 1996; Woo et al., 2001; Stirnberg et al., 2002, 2007; Sorefan et al., 2003; Booker et al., 2004; Ishikawa et al., 2005; Snowden et al., 2005; Zou et al., 2005; Johnson et al., 2006; Arite et al., 2007; Simons et al., 2007; Hamiaux et al., 2012). Other phenotypic changes include reduced flower size and weight, changes in stem diameter, altered cambium growth, adventitious root formation, and hypocotyl elongation (Napoli, 1996; Snowden et al., 2005; Shen et al., 2007; Simons et al., 2007; Agusti et al., 2011; Kapulnik et al., 2011; Ruyter-Spira et al., 2011; Kohlen et al., 2012; Rasmussen et al., 2012b). Current theories suggest SLs are produced in response to nutrient stress to stimulate symbiosis with AM fungi and hence improve nutrient uptake, as well as modifying development to limit growth of branches, increase senescence of leaves and hence focus growth on a single shoot.

Karrikins are highly active seed germination stimulants found in the smoke of burning vegetation (Flematti et al., 2004, 2009; Nelson et al., 2012; Waters et al., 2012b). Their role appears to be to stimulate regrowth after forest fires. Karrikin-insensitive mutants in Arabidopsis (*kai2* and *max2*) have been shown to have reduced germination efficiency, longer hypocotyls, and hooked epinastic cotyledons (Nelson et al., 2011; Waters et al., 2012b). However, unlike MAX2, the role of KAI2 appears to be restricted to the earliest stages of seedling development and *kai2* mutants have no altered branching phenotype.

Both karrikin and SL signal molecules require a single LRR type F-box gene (MAX2) and an α/β hydrolase fold protein (KAI2 or DAD2/D14) for signal transduction (Nelson et al., 2011; Hamiaux et al., 2012; Waters et al., 2012b). It is this hydrolase that is perhaps the most interesting feature, since recent evidence suggests that as well as catalyzing hydrolysis of the hormone signal molecule, the protein is also the hormone receptor (Hamiaux et al., 2012).

## BIOSYNTHESIS OF SL

Genetic and physiological studies identified several genes likely to be involved in the synthesis of SLs. Two carotenoid cleavage dioxygenases (CCD7 and CCD8) were identified in Arabidopsis (MAX3, Booker et al., 1999, 2004; and MAX4, Sorefan et al., 2003) and in other model species used to study branching [petunia; DAD3 (Napoli and Ruehle, 1996; Simons et al., 2007), DAD1 (Napoli and Ruehle, 1996; Snowden et al., 2005); pea; RMS5 (Arumingtyas et al., 1992; Johnson et al., 2006), RMS1 (Arumingtyas et al., 1992; Sorefan et al., 2003); rice; HTD1 (Zou et al., 2006), D10 (Arite et al., 2007)] and in many other plant species including liverworts and mosses (Wang et al., 2011; Delaux et al., 2012). Early studies indicated these enzymes were capable of cleaving carotenoids and could lead to synthesis of SLs (Matusova et al., 2005; Gomez-Roldan et al., 2008; Umehara et al., 2008). Additional genes were genetically identified as involved in biosynthesis: an isomerase from rice (D27; Lin et al., 2009) and a cytochrome P450 oxidase from Arabidopsis (MAX1; Stirnberg et al., 2002; Booker et al., 2005). In recent work, Alder et al. (2012) suggests the SL biosynthetic pathway starts with isomerization of all *trans* β-carotene by D27, CCD7 then cleaves at the 9′-10′ bond to release 9-*cis*-β-apocarotenal and β-ionone, and CCD8 acts on the carotenal product to add three oxygen atoms and rearrange the carotenal into carlactone (**Figure 1D**). Carlactone contains the butenolide ring that is common to both SLs and karrikins and the enol-ether bridge present in natural SLs. Carlactone has also been shown to have biological activity in rice branching and parasitic weed germination assays. Subsequent synthesis of SLs from carlactone might involve the cytochrome P450 enzyme.

The SL biosynthetic pathway includes several of the genes identified using genetic and physiological studies as being involved in regulation of branching by SL, although most genes were identified before the hormone itself was identified. It is possible that additional genes are involved in the synthesis of SLs that have not yet been identified, possibly because of functional redundancy in the pathway or because some enzymatic functions may play essential roles in other pathways. The proposed synthetic pathway also includes all the genes where mutants can be restored to wild-type branching phenotype by grafting mutant scions onto wild-type rootstocks and where the mutant phenotype can be complemented by applying SLs (or analogs) directly to the axillary meristem (Napoli, 1996; Stirnberg et al., 2002; Booker et al., 2005; Simons et al., 2007; Gomez-Roldan et al., 2008; Umehara et al., 2008; Drummond et al., 2009a; Lin et al., 2009; Hamiaux et al., 2012).

## RECEPTION AND SIGNAL TRANSDUCTION OF SLs AND KARRIKINS

Studies of branching have also identified a second class of highly branched mutants that are epistatic to the biosynthetic mutants (hence in the same pathway) and which could not be reverted to wild-type phenotype by grafting or direct application of SL (Beveridge et al., 1996; Booker et al., 2005; Simons et al., 2007; Arite et al., 2009; Drummond et al., 2009a; Hamiaux et al., 2012). This class included *max2*/*ore9*/*pps* in Arabidopsis (Woo et al., 2001; Stirnberg et al., 2002; Shen et al., 2007), *rms4* in pea (Arumingtyas et al., 1992; Johnson et al., 2006), *d14/htd2/dwarf88* in rice (Arite et al., 2009; Gao et al., 2009; Liu et al., 2009), and *dad2* in petunia (Napoli and Ruehle, 1996; Hamiaux et al., 2012). Because these mutants appeared to act locally and are insensitive to SL, it was postulated that they might be involved in signal reception and transduction.

*MAX2* was cloned from Arabidopsis and shown to be an F-box protein and is part of the SCF complex that acts to ubiquitinate target proteins, which are then usually degraded by the proteosome (Stirnberg et al., 2002; Stirnberg et al., 2007). The gene was previously identified as *ore9* in a screen for delayed leaf senescence (Woo et al., 2001) and subsequently identified as *pps* in a screen for altered photomorphogenesis (Shen et al., 2007). *RMS4* is an ortholog of *MAX2* (Johnson et al., 2006) and orthologs have also been identified in rice (*D3*; Ishikawa et al., 2005) and petunia (*PhMAX2A* and *PhMAX2B*; Drummond et al., 2012). Phylogenetic analysis of the large F-box gene family places *MAX2* in the LRR_7 clade (Dharmasiri et al., 2005b; Xu et al., 2009) that includes several genes associated with hormone signal transduction [jasmonate receptor, COI1 (Xie et al., 1998; Xu et al., 2002); auxin receptors; TIR1 (Dharmasiri et al., 2005a; Kepinski and Leyser, 2005), and AFB1, 2, 3, and 5 (Dharmasiri et al., 2005b; Walsh et al., 2006); and ethylene-signaling proteins EBF1 and 2 (Guo and Ecker, 2003; Binder et al., 2007)]. This evolutionary relationship to other receptors suggested that MAX2 might be the receptor for the SL signal itself, or for a compound derived from SLs.

**FIGURE 2:**
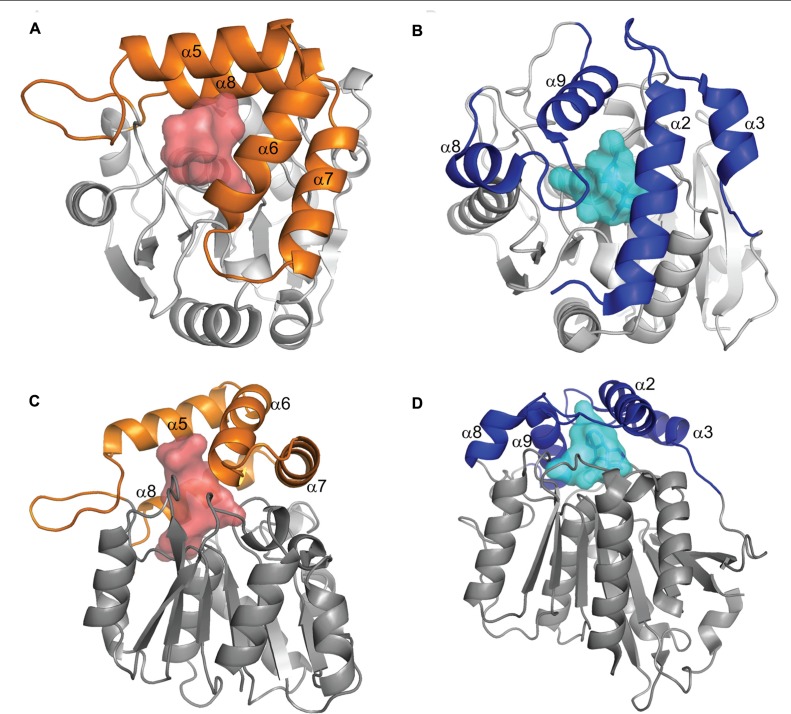
**Comparison of DAD2 and GID1 structures**. Diagrams of the protein structures of DAD2 (left) and GID1 (right) looking down onto the lid **(A,B)** and from the side **(C,D)**. Both proteins have the core α/β hydrolase fold of alternating α helices and β sheets (gray). The proteins have “lids” made up of four α helices (orange for DAD2, blue for GID1) that extend over an internal cavity (shaded, red DAD2, pale blue GID1). Reprinted from Hamiaux et al. (2012), Copyright (2012) with permission from Elsevier.

*D14/HTD2/DWARF88* was cloned from rice (Arite et al., 2009; Gao et al., 2009; Liu et al., 2009) and like the gibberellin hormone receptor *GID1* (Ueguchi-Tanaka et al., 2005) predicted to be an α/β hydrolase fold protein. An ortholog of *D14* has been isolated from Arabidopsis (*Atd14*; Waters et al., 2012b) where it is also involved in branching, and the petunia branching gene *DAD2* has recently also been shown to be an ortholog of *D14* (Hamiaux et al., 2012). The similarity between *GID1* and *DAD2*/*D14* (**Figure 2**) raised the possibility that *DAD2*/*D14* might also act as the receptor for SLs, leaving the signal transduction pathway for SLs unclear, with at least two candidates for the receptor.

The reception and signal transduction pathway for karrikins was examined using a screen for insensitivity to karrikins that identified the gene *KAI2* as required for karrikin signaling (Waters et al., 2012b). *KAI2* (previously isolated as *htl* in a screen for hyposensitivity to light; Sun and Ni, 2011) is also an α/β hydrolase fold protein. Phylogenetic analyses have identified three closely related clades of α/β hydrolase fold proteins, the *D14*/*DAD2* clade, the *KAI2*/*HTL* clade, and a third clade (DAD2-like) with no known functional association as yet (Delaux et al., 2012; Hamiaux et al., 2012; Waters et al., 2012b). The similarity between *KAI2*/*HTL* and *DAD2*/*D14* suggests they may act using the same mechanism. This hypothesis is strengthened by the observation that screens for karrikin insensitivity also identified the F-box protein *MAX2* and showed it was required for karrikin signal transduction (Nelson et al., 2011; Waters et al., 2012a).

The convergence of the karrikin and SL signal transduction pathway on *MAX2* and the close homology between *DAD2*/*D14* and *KAI2*/*HTL* suggest that reception of the two hormones occurs in a similar manner. Two hypotheses have been proposed (Arite et al., 2009) for the role of these α/β hydrolase fold proteins in SL signal transduction:

(1) The hydrolase is an enzyme that converts the inactive mobile compound into an active product that is then perceived by an as yet unknown receptor, possibly *MAX2*;
(2) The hydrolase is itself the receptor of the hormone, by analogy to *GID1*, and signal transduction then proceeds via *MAX2* and the SCF complex.

**FIGURE 3:**
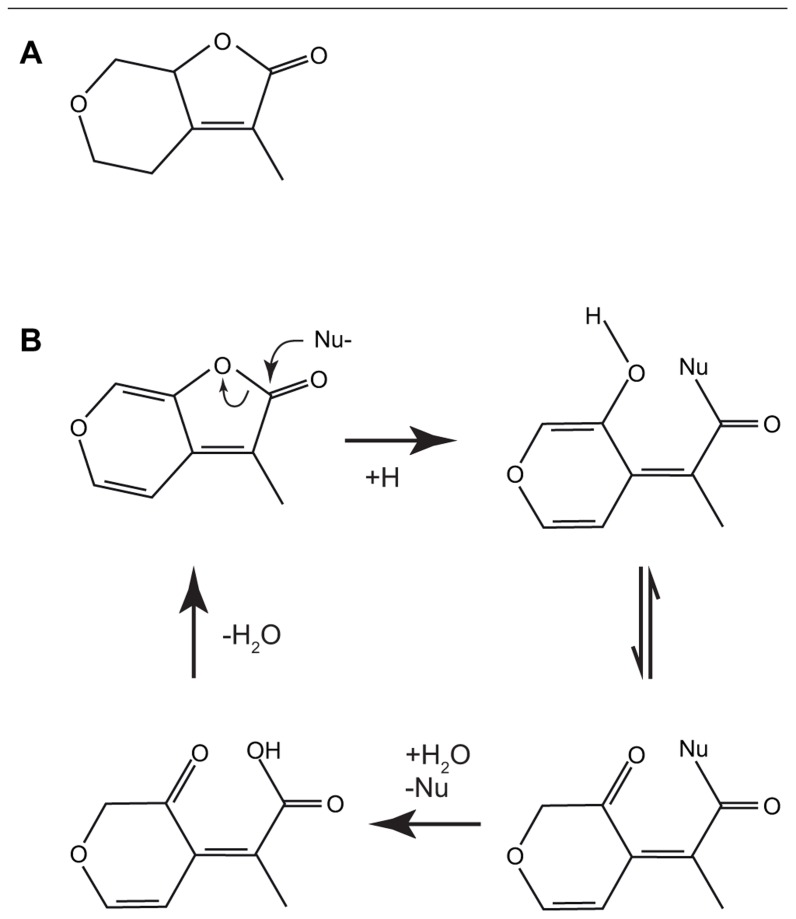
The structures of the biologically active saturated karrikinolide **(A)**. A putative scheme has been proposed (Scaffidi et al., 2012) for the nucleophilic attack of the α/β hydrolase showing the formation of the bound intermediate and the subsequent release to reconstitute the starting karrikin **(B)**.

However, for karrikin signaling the first hypothesis does not seem applicable, since it would appear that if *KAI2* hydrolyses karrikin, the product of the reaction is predicted to have the same structure as the substrate (**Figure 3B**), albeit with an exchange of a water molecule (Scaffidi et al., 2012).

## CLONING AND CRYSTAL STRUCTURE OF DAD2

**FIGURE 4:**
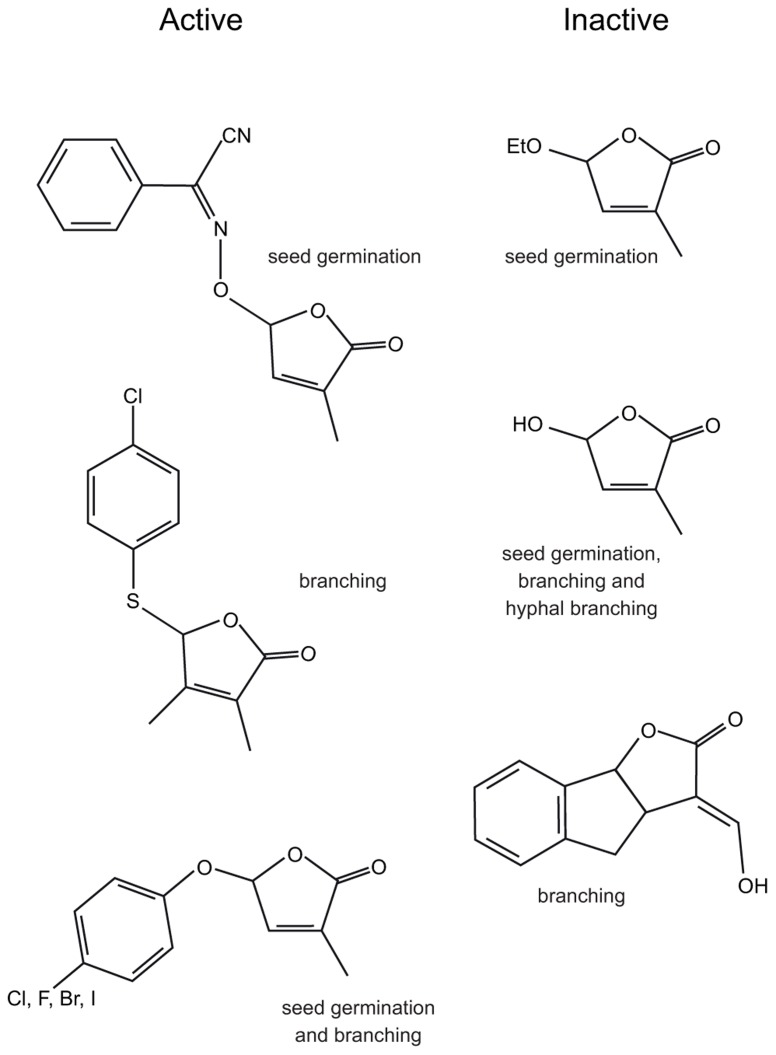
**The structures of some biologically active and inactive SL analogs and the assays used to determine activity**.

The characterization of the *DAD2* gene and its protein has allowed more understanding of the role of the α/β hydrolase fold protein in SL signal transduction. The X-ray crystal structure of DAD2 has been solved and confirms the protein is indeed in the α/β hydrolase fold family (Hamiaux et al., 2012). There is a large internal hydrophobic cavity capable of accommodating SLs. The canonical catalytic triad amino acids are all present, although the active site serine (S96) is rotated approximately 30^°^ out of optimal alignment; this may be because the protein was crystallized in an inactive conformation or may have an effect on catalytic activity. DAD2 has been shown to be catalytically active, capable of hydrolyzing the SL analog GR24 into two fragments, one of which is the formyl tricyclic lactone (the ABC rings, shown in **Figure 4**), suggesting cleavage can occur at the enol-ether bridge between the C and D rings (**Figure 1A**). However, turnover appears to be slow, with 50% hydrolysis of a 20 molar excess of GR24 taking 3 h *in vitro* (Hamiaux et al., 2012). This enzyme activity suggests that DAD2 might process the hormone rather than acting as the receptor itself.

Protein stability studies showed that DAD2 becomes less stable in the presence of GR24, indicating a change in protein conformation. Yeast two-hybrid studies showed DAD2 was able to interact with a petunia ortholog of MAX2 (PhMAX2A) in a GR24 concentration-dependent manner with an apparent *K*_d_ of 360 ± 50 nM (Hamiaux et al., 2012). However, the DAD2-mediated cleavage products of GR24 were unable to elicit either conformational change in DAD2 or interaction with PhMAX2A, providing evidence that the role of DAD2 in signal transduction is not simply cleavage of SLs to produce an active product.

The interaction between DAD2 and PhMAX2A is very interesting and suggests that in the presence of GR24, DAD2 changes conformation and becomes able to bind to the F-box protein and hence the SCF complex. Such a hormone-dependent interaction has similarities to gibberellin (GA) reception by GID1 and suggests a mechanism for signal transduction that involves SCF-mediated ubiquitination of target proteins. The ability to bind the mobile signaling molecule, change conformation and then interact with a putative signal transduction protein suggests DAD2 is the hormone receptor.

If DAD2 is a receptor, what then is the role of the hydrolytic activity? Several authors have suggested that the hydrolase could convert a biologically inactive transported compound into an active product at the site of action. However, studies have shown that the products of hydrolysis of the SL analog GR24 are unable to induce germination of parasitic weeds (Zwanenburg et al., 2009), hyphal branching in AM fungi (Akiyama et al., 2010), and most importantly do not inhibit branching (Boyer et al., 2012; Hamiaux et al., 2012). Since the products of hydrolysis of SLs appear to be inactive, it is unlikely that DAD2 acts simply as an enzyme to produce a biologically active product. Another possibility is that the catalytic activity exists to allow removal of the signal molecule from the receptor, allowing the receptor to be recycled into an active state as well as destroying the active signal molecule. A model that does not exclude the role of recycling the receptor is that the formation of an intermediate, after nucleophilic attack of the active site serine on the hormone, either requires or results in a conformational change that in turn mediates signal transduction.

Initial investigations of the role of catalytic activity in signal transduction used mutation of either the active site serine or histidine to an alanine (DAD2S96A and DAD2H246A) to abolish hydrolytic activity (Hamiaux et al., 2012). Active site mutants were slightly less stable than the wild-type protein but unlike the wild-type protein showed no change in stability in response to GR24, suggesting they do not interact with GR24. The DAD2S96A protein was also unable to interact with PhMAX2A in the presence of GR24 or in the presence of the hydrolysis products of GR24, suggesting the products of GR24 cleavage do not directly bind with the F-box and subsequently cause interaction with DAD2. The DAD2S96A protein was also unable to complement the *dad2* mutant in transgenic plants. These observations suggest that hydrolysis is integral to signal transduction, although it is possible that the serine and/or histidine are integrally involved in SL binding and hence the active site mutants are simply unable to bind the hormone.

Comparison of DAD2 to GID1 highlighted the fact that both proteins have a “lid” consisting of α helices over the hydrophobic cavity (**Figure 2**; Hamiaux et al., 2012). In the case of the non-catalytic receptor GID1, interactions between GA and the “active site serine and aspartic acid” appear to result in movement of the lid to close over the binding site (Murase et al., 2008; Shimada et al., 2008). Subsequent interactions with both the DELLA proteins, which stabilize the GID1 GA interaction (Ueguchi-Tanaka et al., 2005, 2007; Nakajima et al., 2006; Willige et al., 2007; Murase et al., 2008; Ueguchi-Tanaka and Matsuoka, 2010) and the SCF complex lead to degradation of the DELLA proteins which also have a role in binding and repression of specific transcription factors (Shimada et al., 2008; Wang et al., 2009; Hirano et al., 2010; Gao et al., 2011; Hauvermale et al., 2012).

**FIGURE 5:**
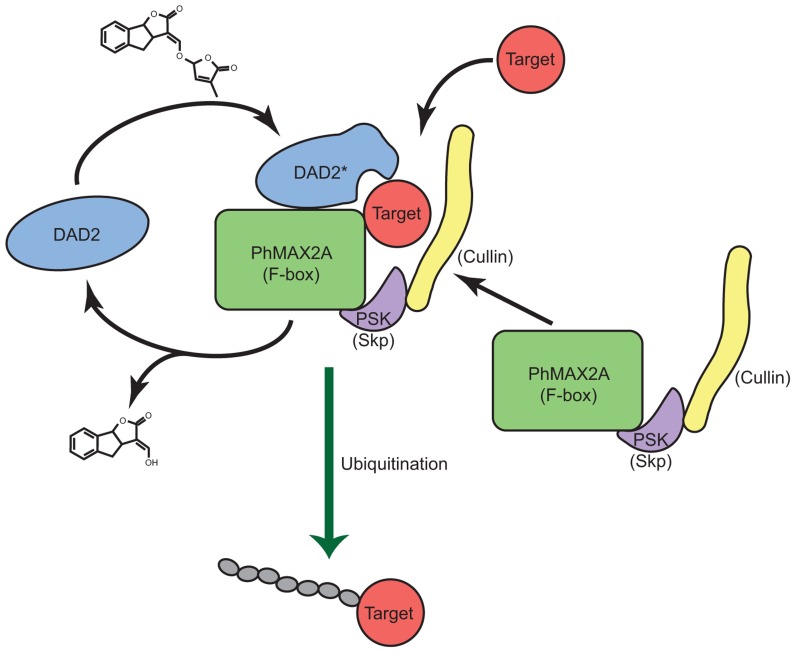
**A model for strigolactone reception**. A model for the reception and signal transduction of the SL signal by the α/β hydrolase DAD2 is shown. DAD2 binds and reacts with SL, changing conformation to form DAD2*. DAD2* interacts with the F-box protein PhMAX2A and the other partners of the SCF^MAX2^ complex. Target protein(s) are recognized by the DAD2-SCF^MAX2^ complex and ubiquitinated. DAD2* hydrolyses SL, releasing the products of hydrolysis. DAD2 disengages from the SCF^MAX2^ complex returning to its original conformation allowing it respond to fresh SL signal.

Based on structural similarities between GID1 and DAD2 and the observed *in vitro* interaction of DAD2 with PhMAX2A, as well as the requirement of MAX2 in karrikin signaling, it is tempting to suggest SL and KAR signal reception might be mediated by binding of SLs and karrikins by the α/β hydrolase fold protein, followed by interaction with MAX2 and the SCF complex and signal transduction, then by degradation of downstream signaling protein(s) (**Figure 5**).

One candidate for a direct target of SL-induced degradation is the LIF gene from petunia (Nakagawa et al., 2005), which is a promoter of branching and part of the superman family of TFs. However, the lack of knockout phenotypes for the LIF gene and the absence of orthologs in other model systems have limited the study of this gene. The TCP class of proteins (Martin-Trillo and Cubas, 2010) including TB1 from maize (Doebley et al., 1997) and the Arabidopsis homologs, BRC1 and BRC2 (Aguilar-Martínez et al., 2007; Finlayson, 2007) are also potential targets of a SL-DAD2-activated SCF^MAX^^2^ complex; however, many TCP genes act as repressors of growth (Martin-Trillo and Cubas, 2010), and hence would seem to be unlikely to be direct targets of SL-induced degradation, although some TCP genes can act as promoters of growth (Bai et al., 2012). The observation that blocking protein synthesis results in an increase in transcript levels of the pea homolog of BRC1 (Dun et al., 2012) suggests the presence of a repressor that is turned over at a significant rate, such a repressor of a repressor of growth could be a target for SCF^MAX2^-mediated degradation.

As yet, no candidates for downstream targets of karrikin signaling have been identified. While some overlap with SL signaling is possible since they both involve the same F-box gene, it is worth noting that *kai2* mutants do not have an altered branching phenotype (Waters et al., 2012b), this suggests the signal transduction pathways diverge at some point. While some of the difference in phenotype may be due to differential expression patterns of the receptor proteins it is also possible that recognition of downstream targets is not solely specified by MAX2, with DAD2 and KAI2 directly involved in interactions with target proteins, or that interactions between the α/β hydrolase fold proteins and the F-box protein are able to alter the specificity of the SCF^MAX2^ complex.

## BIOLOGICAL ASSAYS FOR STUDYING SL AND KARRIKIN ACTIVITY

Several studies have examined the chemistry and biology of SL and karrikin analogs. These structure activity relationship (SAR) studies show many different analogs can mimic SL function in parasitic weed germination (Mangnus et al., 1992; Mangnus and Zwanenburg, 1992; Nefkens et al., 1997; Kondo et al., 2007; Yoneyama et al., 2009, 2010; Zwanenburg et al., 2009; Xie et al., 2010; Fukui et al., 2011; Mwakaboko and Zwanenburg, 2011; Zwanenburg and Mwakaboko, 2011), hyphal branching of AM fungi (Akiyama et al., 2005, 2010), and most recently in branching of rice (Fukui et al., 2011) and pea (Boyer et al., 2012). Several studies have also shown that SLs, and a fluorescent SL analog, can alter the growth of roots and these may prove useful in the development of biological assays (Kapulnik et al., 2011; Ruyter-Spira et al., 2011; Kretzschmar et al., 2012; Rasmussen et al., 2012a). The biological activity of karrikin analogs has been measured in seed germination studies (Flematti et al., 2007, 2010; Scaffidi et al., 2012; Waters et al., 2012c) and identified several features of the molecule that are important to activity.

## KARRIKIN SAR IN GERMINATION

Compared with naturally occurring SLs, karrikins are relatively simple molecules (**Figure 1B**) comprising a butenolide fused to a pyran ring. This has allowed several important features of the interactions between signal and biological response to be identified (Flematti et al., 2007, 2010). Modification of the pyran ring was possible at several sites without abolishing activity, including the addition of bulky side groups at the C5 position on the pyran ring that suggests that the binding pocket for karrikins is either flexible or larger than required for karrikin itself. However, the pyran ring was essential for activity. It had been suggested that the presence of a Michael acceptor site on the pyran ring might be important for activity, however a karrikin analog with a saturated pyran ring and no Michael acceptor at the 5 or 7 position remained biologically active (**Figure 3A**; Flematti et al., 2010; Scaffidi et al., 2012). Modification at the C3 position on the butenolide ring showed that bulky or electron-withdrawing groups reduced activity, whereas the electron-donating methyl group increased activity (Flematti et al., 2007, 2010).

These observations led to a model where KAI2 binds karrikins and karrikin analogs by nucleophilic attack on the butenolide ring at the carbonyl atom (**Figure 3B**; Scaffidi et al., 2012; Waters et al., 2012c). This would lead to the formation of an intermediate where KAI2 is covalently bound to karrikin via the active site serine. Subsequent hydrolysis results in release of the signal molecule; however, the original molecule is regenerated in this reaction. If this model is correct, then the substrate and product of the catalysis are the same. A corollary of this model is that KAI2 is likely to be the receptor for the karrikin signal and not an enzyme that processes karrikin into a molecule that is received elsewhere. Modifications of the pyran ring including substitution of O6 with an N do not abolish activity. However, analogs that are not targets for nucleophilic attack at the butenolide ring are not germination stimulants. These data strongly suggest that for KAI2 reception of karrikins it is the nucleophilic attack at the carbonyl atom of the butenolide ring that is important in signal transduction.

## SL SAR IN PARASITIC WEED GERMINATION AND HYPHAL BRANCHING

For parasitic weed germination assays, the receptor for SLs is likely to be a homolog of DAD2 and presumably when these important parasitic weeds are fully sequenced it will be possible to identify the receptor. However, for hyphal branching assays, the fungal receptors are as yet unknown and presumably SL targets a receptor that is different from that targeted in branching. However, the similarities of the signal molecules suggest that reception of the signal broadly follows a similar mechanism and the findings of these studies are supported by SAR studies using branching in pea and rice, albeit with some differences in sensitivity in the different biological systems, which presumably reflects specific differences between the receptors (Fukui et al., 2011; Boyer et al., 2012).

Initial SAR studies for SL analogs were focused on stimulation of seed germination of parasitic *Orobanche* and *Striga* species (Mangnus et al., 1992; Mangnus and Zwanenburg, 1992; Nefkens et al., 1997; Kondo et al., 2007; Yoneyama et al., 2009, 2010; Zwanenburg et al., 2009; Xie et al., 2010; Fukui et al., 2011; Mwakaboko and Zwanenburg, 2011; Zwanenburg and Mwakaboko, 2011; Kgosi et al., 2012). These studies showed that alterations of the A, B, and C rings, including deletions of the entire A and B rings, while able to modify activity, did not necessarily abolish activity. However, the D ring alone was not biologically active (**Figure 4**; Zwanenburg et al., 2009; Akiyama et al., 2010). This led to the hypothesis that biological activity depended on the D ring and the presence of a suitable Michael acceptor, usually the enol-ether bridge between the C and D rings (Xie et al., 2010). SAR studies using hyphal branching of AM fungi to assay biological activity also suggested the D ring was important (Akiyama et al., 2010). However, studies in both seed germination (Kondo et al., 2007; Mwakaboko and Zwanenburg, 2011; Zwanenburg and Mwakaboko, 2011; Kgosi et al., 2012) and hyphal branching (Akiyama et al., 2010) suggested that the enol-ether bridge was not essential and the carbon–carbon double bond could be replaced by a carbon-nitrogen double bond (**Figure 4**; Kondo et al., 2007; Akiyama et al., 2010). These analogs are not substrates for a simple Michael addition and suggest a different mechanism could be involved.

## SL SAR IN BRANCHING

Two recent studies have used inhibition of branching in rice (Fukui et al., 2011) and pea (Boyer et al., 2012) to assay for biological activity of SL analogs. These studies confirmed many of the observations in seed germination and hyphal branching assays. The first observation from these studies is that it is possible to extensively modify the A and B rings or remove them entirely and still retain activity, this suggests that the signal reception and transduction system does not form strong interactions with the A and B rings. Either the binding pocket is flexible with respect to the surfaces that interact with the A and B rings, or the interactions formed are not significant for biological activity.

By contrast, the butenolide D ring is required for biological activity. Electron-donating groups such as CH_3_ at the C3′ or C4′ position (**Figure 1A**) enhance biological activity, whereas electron-withdrawing groups reduce activity, suggesting that it is the chemical interaction at the butenolide ring that is significant in signal transduction.

In addition to showing that the A, B, and C rings could be heavily modified, studies in rice (Fukui et al., 2011) and pea (Boyer et al., 2012) both showed that the enol-ether bridge between the C and D rings or the presence of a Michael acceptor between the C and D rings was not required for biological activity. Instead, both of these studies identified that biologically active compounds comprise a butenolide D ring and a good leaving group at the C2′ position of the D ring (**Figure 4**).

## SAR STUDIES SUGGEST A MODEL FOR SIGNAL RECEPTION

These observations from SL and karrikin SAR studies combine to suggest a model of hormone reception that depends on nucleophilic attack on the butenolide ring by the receptor/enzyme at the carbonyl atom. Modifications that promote nucleophilic attack appear to enhance biological activity, whereas modifications that prevent or inhibit nucleophilic attack abolish activity. This would result in an intermediate where the hormone is covalently bound to the receptor/enzyme. In most SL analogs, release of the hormone appears to occur by hydrolysis at an enol-ether bridge between the D ring and the remainder of the molecule, or in the pyran ring for karrikins, but for some analogs a different leaving group can substitute. For SL analogs, the presence of a more effective leaving group at C2′ of the butenolide D ring increases biological activity.

This model of binding by nucleophilic attack at the butenolide ring suggests the important interaction between the hormone and the receptor/enzyme is not stabilized by multiple interactions with the entire binding pocket, but instead is a covalent interaction where part of the molecule (the butenolide ring) reacts with the receptor. The formation of a covalently bound intermediate could require, or result in, a change in receptor conformation that in turn leads to signal transduction. However, it is clear from SAR studies of SL analogs that while considerable variability is possible in the A, B, and C rings, some specificity must be present in this portion of the molecule, particularly because karrikin does not appear to activate DAD2/D14, since *kai2* hypocotyls are insensitive to karrikin but remain sensitive to GR24 (Waters et al., 2012b).

## A COMBINED MODEL FOR SIGNAL RECEPTION

The combined observations from SAR studies of SL analogs and karrikin analogs and the observation that the catalytic triad is required for DAD2 activity suggest a model for signal binding that involves nucleophilic attack by the protein (which requires an intact catalytic triad) on the carbonyl group of the butenolide ring. Modifications of either the protein or the signal molecule that prevent that nucleophilic attack inhibit or abolish bioactivity. A transition state intermediate or a stable intermediate with the hormone covalently bound to the receptor/enzyme is formed and a conformational change occurs in the protein, likely to involve the four α helices of the “lid.” The conformational change allows interaction with the F-box protein (MAX2) which results in the ubiquitination of downstream signal transduction proteins (**Figure 5**).

This model is consistent with all the current observations for SL and karrikin signal transduction, including the SAR studies and the observed GR24-induced conformational changes in DAD2 and interactions between DAD2 and PhMAX2A. This model has similarities to GA reception by GID1, including the close association of the “active site serine and aspartic acid” with GA and interactions with the SCF complex. However, this model for signal reception is unusual in the requirement for catalytic activity by the receptor/enzyme and will need further confirmation, but for now it forms a framework for further study of the signal transduction pathway.

## IMPLICATIONS FOR RECEPTION OF THE SL SIGNAL BY PARASITIC WEEDS AND AM FUNGI

Both SL and karrikin reception appear to involve the nucleophilic attack on the hormone by a catalytically active α/β hydrolase fold protein. Since analogs of SL are able to stimulate seed germination by parasitic weeds, this suggests that the receptor for this signal might also be a catalytically active α/β hydrolase fold protein, possibly an ortholog of DAD2. However, for AM fungi the SL receptor is unlikely to be an ortholog of DAD2, since phylogenetic analysis has not found a DAD2 ortholog in fungi (Delaux et al., 2012; Hamiaux et al., 2012; Waters et al., 2012b), yet the receptor is still likely to be a catalytically active α/β hydrolase fold protein. While sequence-based searches of the, as yet limited, genome databases for these organisms has not revealed a DAD2 or KAI2 homolog, it is possible that a different α/β hydrolase fold protein has adapted to this role, particularly in AM fungi. Because α/β hydrolase fold proteins are not well conserved at the primary sequence level, it is not surprising that searches thus far would not have highlighted the receptor. A structure-based search may well reveal possible candidates. However, as shown by comparisons of rice tillering or pea branching assays with parasitic weed germination assays, using the same SL analogs (Fukui et al., 2011; Boyer et al., 2012), there are differences in sensitivity to SL analogs that suggest there will be differences between the receptors. If the receptors have significant differences, this may allow the design of compounds that can alter traits specifically, for example improving symbiosis with AM fungi without stimulating parasitic weed germination or altering branching.

## EVOLUTION OF THE RECEPTOR

The α/β hydrolase fold proteins are present in at least 89 different family groups, probably related by divergent and/or convergent evolution (Heikinheimo et al., 1999; Carr and Ollis, 2009). They encompass a wide range of diverse enzymes (peptide hydrolases, lipases, esterases, haloperoxidases, dehalogenases, and C-C bond breaking enzymes; Holmquist, 2000), as well as a plant hormone receptor (GID1) without enzyme activity. Members of the family also include enzymes involved in the turnover of hormones and signaling molecules such as acetylcholine esterase in neuron signaling and juvenile hormone esterase in insect development (Holmquist, 2000).

It has been suggested that GID1 evolved from the closely related hormone-sensitive lipases (HSLs) by adaptation of amino acids in the lid to interact with GA as well as loss of the active site histidine and consequent loss of enzyme activity (Shimada et al., 2008). The GID1 receptor has structural similarities to the HSLs, with four α helices from two loops folding over the active site. In the binding pocket GID1 has strong interactions between GA and both the nucleophilic serine and the aspartic acid similar to those of the catalytic triad in HSLs (Shimada et al., 2008), suggesting GID1 evolved from an active enzyme. Mutations made at amino acids of GID1 that were conserved in plants but not amongst the HSLs showed that these positions were important for GA binding, suggesting these amino acids were involved in the evolution of the receptor from the catalytically active HSL structure.

While DAD2 and KAI2 have a similar topology to the HSLs, they lack one N-terminal β strand and any N-terminal α helices, and the α helices that comprise the lid derive from a single loop rather than the two loops as in the HSLs. The DAD2 structure is classified as in the RBSQ-like sub-family and part of the larger Abhydrolase_6 family (Hotelier et al., 2004) along with other α/β hydrolase fold proteins that do not obviously fall into one of the more defined structural families. This lack of strong structural similarity to other members of the fold family, combined with the very weak primary sequence similarity typical of α/β hydrolase fold proteins, makes it difficult to identify a likely progenitor for the evolution of DAD2. As more structures are determined in this family, it may become easier to identify related enzymes and also identify conserved amino acids that may be important in the evolution of DAD2 or KAI2.

**FIGURE 6:**
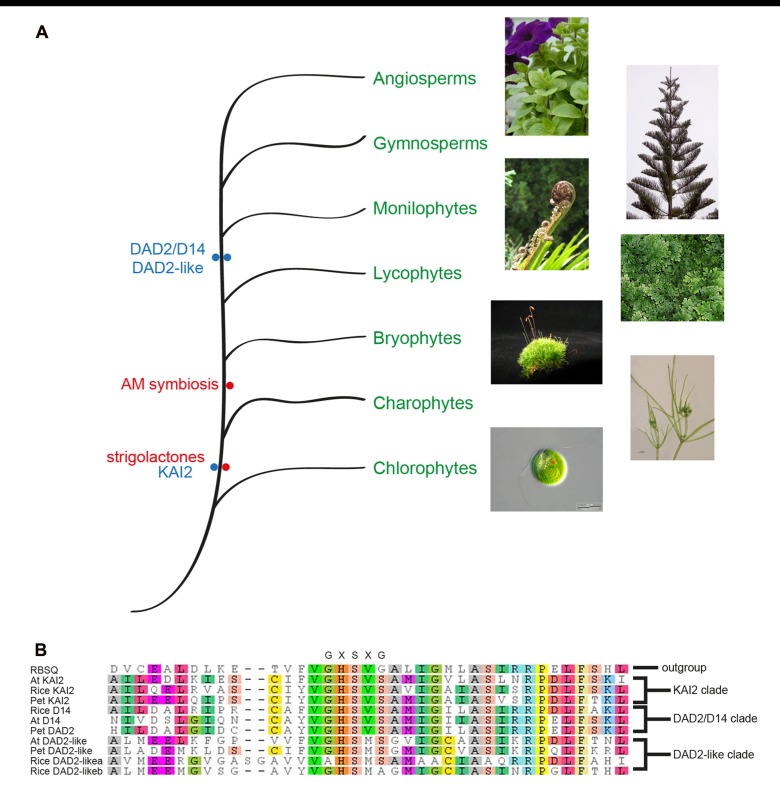
Phylogenetic representation of the plant kingdom showing the major lineages and the observation of SL presence and the DAD2 and KAI2 genes in ancestral lineages **(A)**. Photographs of Petunia, Norfolk pine, Black Ponga, and moss are authors own; Nitella image from http://www.nybg.org/science/conservation.php by Dr. Kenneth Karol; *Chlamydomonas* image from http://www.protisten.de/english/index.html by Wolfgang Bettighofer; *Selaginella* image from http://en.wikipedia.org/ wiki/File:Selaginella-sp.jpg by Luis Fernández García. A portion of a protein sequence alignment using Muscle (Edgar, 2004) of the Arabidopsis, rice, and petunia members of the three DAD2/D14/KAI2 clades with RBSQ as the outgroup (Drummond et al., 2010). The nucleophilic elbow GXSXG motif at the active site serine is shown above the alignment **(B)**. RSBQ, Acc. No. 16080463; At KAI2, Acc. No. 15235567; Rice KAI2, Acc. No. 115453689; Pet KAI2, ABHF_7407 (from ChromDB http://www.chromdb.org/index.html); Rice D14, 32980850; At D14 Acc. No. 18396732; Pet DAD2, ABHF_7401; At DAD2-like, 15230110; Pet DAD2-like, ABHF_7407; Rice DAD2-likea, 115465775; Rice DAD2-likeb, 115438152.

One of the canonical features of α/β hydrolase fold proteins is the GXSXG motif around the nucleophilic serine, where the glycines are non-ramachandran amino acids that form the nucleophilic elbow. Phylogenetic analysis of genes similar to DAD2 revealed three closely related clades (Delaux et al., 2012; Hamiaux et al., 2012; Waters et al., 2012b). In all three clades the nucleophilic elbow differs from the canonical motif and is GHSVS in the DAD2 and KAI2 clades and is GHSMS in the closely related DAD2-like clade (**Figure 6**). Whether the altered motif has any biochemical or biological significance is still to be determined. It is possible that the altered nucleophilic elbow reduces the rate of catalysis, allowing these genes to function as receptors.

Of the three clades, only the KAI2 clade has representatives from* Selaginella moellendorffii*, *Physcomitrella patens*, *Marchantia polymorpha*, and *Nitella mirabilis* (Delaux et al., 2012; Hamiaux et al., 2012; Waters et al., 2012b), suggesting this may be the ancestral clade and the other clades may have been formed by gene duplication after the divergence from mosses and liverworts, followed presumably by specialization of functions (**Figure 6**). The absence of a DAD2 ortholog in the ancestral lineages is interesting since representatives of these lineages have been shown to produce SLs and respond to SLs (even though the ancestral CCD8 ortholog is significantly different from CCD8 in higher plants and may not be functional; Proust et al., 2011; Delaux et al., 2012). The observation that *Nitella* species both produce SLs and have orthologs of KAI2 is interesting since this lineage diverged before the appearance of AM fungi (**Figure 6**), which suggests SLs had a role in plants prior to being involved in symbiosis with AM fungi (Delaux et al., 2012). A role for SLs in rhizoid elongation has been shown (Proust et al., 2011; Delaux et al., 2012) and it will be interesting to see if the KAI2 ortholog in these species can act as the SL receptor.

As yet, no role has been identified for genes in the DAD2-like clade. Both the DAD2 and KAI2 clades have representatives from monocots, and eudicots and for the DAD2 clade the role is conserved in monocots and eudicots. However, the DAD2-like clade is less conserved. The monocot genes most similar to DAD2-like cluster as a separate group with some sequence differences around the nucleophilic elbow. The eudicot members of the DAD2-like clade show more sequence variation than is seen for the other two clades. Whether this sequence variation is associated with variation in function or if there are differences in function between monocot and eudicot DAD2-like genes remains to be determined.

Given the sequence similarity between DAD2-like, DAD2 and KAI2, combined with the lack of the canonical GXSXG motif around the nucleophilic serine, it seems reasonable to predict that DAD2-like would have weak catalytic activity and would act as the receptor for an as-yet unknown signal compound. Furthermore, based on structural similarities between the karrikins and the SLs, it seems likely that this signal molecule would contain a butenolide ring with an effective leaving group.

It is tempting to see the KAI2/DAD2 receptor/enzyme as an intermediate step in the evolution of a pure receptor like GID1, where an enzyme takes on a role in a signal transduction pathway, perhaps in signal turnover, and initially loses catalytic efficiency before losing the catalytic activity entirely. While purely speculative, such a hypothesis for the evolution of a receptor from an enzyme is consistent with the observed roles of DAD2 and KAI2.

## FUTURE DIRECTIONS

The characterization of the structure of DAD2 and discovery of the interaction between DAD2 and the F-box protein MAX2 provides a major step forward in the understanding of SL signal transduction. The similarities between karrikin and SL signal transduction and the SAR studies with both SLs and karrikins suggest a model for hormone reception that involves reception by an active enzyme, which leads to interaction with the SCF complex and presumably degradation of downstream target proteins.

Several features of this model remain to be understood. The exact role of catalysis in signal reception is not certain; does conformational change in DAD2/D14 occur as a consequence of binding or is the conformational change needed before binding can occur? Is hydrolysis of SLs absolutely required or can analogs be designed that can activate the pathway without being cleaved? How are the SL receptors in parasitic weed species and AM fungi related to DAD2 and KAI2? Which surfaces of the receptor are involved in the interaction with MAX2? MAX2 appears to be able to distinguish between signals from DAD2 and KAI2 to activate different response pathways leading to different phenotypic changes; how is the differentiation achieved? What are the downstream targets of karrikin and SL signaling? The recent advances in understanding of both SL and karrikin signal transduction have set the stage for future progress in this field.

## Conflict of Interest Statement

The authors declare that the research was conducted in the absence of any commercial or financial relationships that could be construed as a potential conflict of interest.
